# Is arterial ductal stent as effective as surgical shunt for palliation in staged repair of tetralogy of Fallot with pulmonary stenosis?

**DOI:** 10.1093/icvts/ivaf088

**Published:** 2025-04-21

**Authors:** Yan Le Ho, Maruti Haranal, Marhisham Che Mood, Esther Mathias Ajik, Nurul Fazira Basran, Mazeni Alwi, Sivakumar Sivalingam

**Affiliations:** Department of Cardiothoracic and Vascular Surgery, National Heart Institute, Kuala Lumpur, Malaysia; Department of Paediatric Cardiac Surgery, UN Mehta Institute of Cardiology and Research, Ahmedabad, India; Paediatric and Congenital Heart Centre, National Heart Institute, Kuala Lumpur, Malaysia; Paediatric and Congenital Heart Centre, National Heart Institute, Kuala Lumpur, Malaysia; Clinical Research Department, National Heart Institute, Kuala Lumpur, Malaysia; Paediatric and Congenital Heart Centre, National Heart Institute, Kuala Lumpur, Malaysia; Department of Cardiothoracic and Vascular Surgery, National Heart Institute, Kuala Lumpur, Malaysia

**Keywords:** tetralogy of Fallot with pulmonary stenosis, staged repair of Tetralogy of Fallot, modified Blalock-Taussig-Thomas Shunt, arterial ductal stent, pulmonary annular growth, pulmonary valve sparing repair

## Abstract

**INTRODUCTION:**

A staged repair strategy in the form of Modified Blalock-Taussig-Thomas shunt has been performed to facilitate the growth of pulmonary valve annulus, so that patients with marginally small annulus could benefit from pulmonary valve-sparing repair. However, little has been reported on the influence of arterial ductal stent (ADS) on the growth of pulmonary annulus and pulmonary artery, with subsequent valve-sparing repair.

**METHODS:**

Patients who underwent staged repair of tetralogy of Fallot with Pulmonary Stenosis with either ADS or surgical shunt were included. Echocardiographic and angiographic measurements of pulmonary annulus and pulmonary artery prior to initial palliation and complete repair were recorded.

**RESULTS:**

A total of 110 patients were included, 44 (40%) patients underwent ADS and 66 (60%) patients had surgical shunt. Pulmonary annulus and pulmonary arteries grew significantly following palliation with both ADS (*P* = 0.011) and surgical shunt (*P* < 0.01), with a similar rate of increment (*P* = 0.205). There was no significant difference in the rate of valve-sparing repair between the 2 groups (MBTTS, 62.1% vs ADS, 47.7%, *P* = 0.149). However, patients who underwent ADS had shorter stays in hospital (*P* = 0.048). Reintervention rate and mortality rate in the interstage period were similar in both groups (*P* = 0.229 and *P* = 0.210, respectively). There was no reintervention in patients who successfully underwent valve-sparing repair following both palliation groups in the follow-up period.

**CONCLUSIONS:**

ADS is as effective as surgical shunt as a palliative procedure in promoting the growth of pulmonary annulus and pulmonary arteries, with comparable rate of valve-sparing repair during corrective surgery.

## GRAPHICAL ABSTRACT

**Figure:**
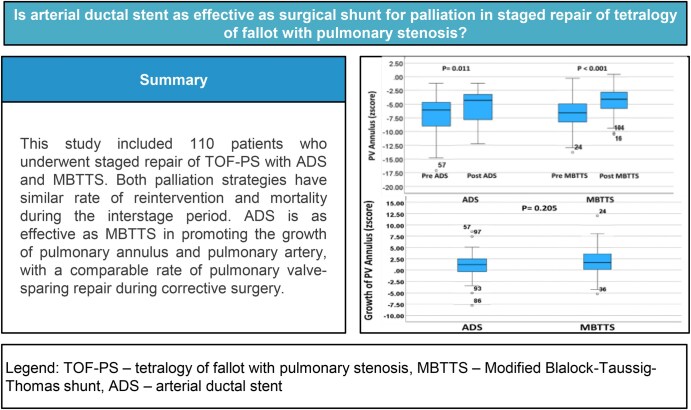

## INTRODUCTION

Although the history of tetralogy of Fallot with pulmonary stenosis (TOF-PS) repair extends over 6 decades, the optimal surgical strategy of corrective surgery has remained controversial and is still under debate. The challenge of early exposure to cardiopulmonary bypass and its complications inadvertently increases the early morbidity and mortality associated with early neonatal TOF-PS repair [1]. Moreover, transannular patch (TAP) reconstruction of the right ventricular outflow tract (RVOT) was reported to be associated with possible deleterious effects of long-term pulmonary regurgitation (PR) leading to adverse physiological sequelae such as RV dilation, dysfunction and mortality [2]. Therefore, some have advocated palliating symptomatic neonates with either modified Blalock-Taussig-Thomas shunt (MBTTS), arterial ductal stent (ADS) or RVOT stent to allow outgrowth of pulmonary valve annulus (PVA) over its somatic growth, so that patient with marginally small annulus could benefit from pulmonary valve sparing repair (VSR) during final TOF correction [1, 3]. To date, the evidence supporting the growth of PVA and pulmonary arteries (PA) with the subsequent VSR after the initial palliation is still limited to patients undergoing surgical shunt, since MBTTS has been the preferred procedure for palliation [4, 5]. literature describing the impact of ADS on the growth of PVA and PA in patients with TOF-PS is very scarce. Furthermore, there is no data comparing whether the rate of growth of PVA and PA after ADS is comparable to that of surgical shunt in a staged repair strategy. Thus, this study aimed to evaluate the rate of growth of PVA and PA after an initial palliative procedure with either ADS or MBTTS in patients diagnosed of TOF-PS, and whether the resultant rate of pulmonary valve-preserving TOF repair after both palliative procedures are comparable to each other.

## PATIENTS AND METHODS

### Patients

This study was approved by Institutional Review Board on 20/03/2024 with the Registration ID of IJNREC/663/2024. This is a single-institution retrospective observational study conducted at the National Heart Institute, Malaysia. The study cohort included all patients with a diagnosis of TOF-PS who underwent staged repair with an initial palliative procedure of either ADS or MBTTS from 2014 to 2022. Patients who underwent primary TOF repair, and patients diagnosed of TOF with pulmonary atresia with or without major aorto-pulmonary collateral arteries were excluded. Patients with missing or incomplete data and follow-up were also excluded. Data are categorized into 2 groups depending on the initial palliative procedure: ADS group and MBTTS group. Baseline demographic characteristics and surgical profiles at initial palliation and complete TOF repair were recorded. The procedural outcomes of both techniques including the total length of hospital and intensive care unit (ICU) stay, reintervention rate and mortality were analysed and recorded. Patient consent was waived as data was collected retrospectively from medical record system and all personal details are anonymized. Primary end-points were rate of growth of PVA and PA after initial palliation, and the subsequent rate of valve-sparing repair between both palliation groups. The secondary end-point measures the rate of reintervention and mortality between both groups.

### Modified Blalock-Taussig-Thomas shunt

MBTTS was constructed using polytetrafluoroethylene(Gore-Tex) graft with predetermined diameter, either through a median sternotomy or thoracotomy. The size range between 3 and 5 mm were used depending on the weight of patients. When performed through a median sternotomy, clinical condition of the patient dictates the need for cardiopulmonary bypass.

### Arterial ductal stenting

In cardiac catheterization, baseline measurements of PA and PVA, with arterial duct morphology were examined and recorded. The details of this procedure are described in our previous study by Alwi M *et al.* [6]. ADS is preferred over MBTTS in cyanotic patients with patent arterial duct, which is responsive to the vasodilating effect of prostaglandin E2 infusion, as well as arterial ducts with favourable morphology without significant branch PA stenosis.

### Follow-up during interstage period (pre-palliation vs pre-complete repair by echocardiographic measurement)

Patients were routinely followed up for clinical assessment, echocardiography and cardiac catheterization following palliation. Serial echocardiography was performed in all patients after the initial palliative procedure by an experienced senior echocardiographer. The absolute diameter of PVA was measured and recorded at 2 occasions: before the palliative procedure and before the final TOF correction. Pulmonary annular size was normalized by the body surface area and evaluated using a *Z* score. In cardiac catheterization, the size of the PA was evaluated with the pulmonary arterial index was developed by Nakata [7]. The changes before and after the palliative procedure were compared. In the surgical shunt group, the pulmonary artery index before the palliation was examined with echocardiography because most of the patients underwent a surgical shunt in the neonatal period without cardiac catheterization.

### Complete correction of tetralogy of Fallot

At the final correction, the procedure is considered as VSR if a simple valvotomy, commissurotomy or patch pasty of the native pulmonary valve was performed. Whereas a transannular incision with or without a monocusp valve is considered as non-annulus preserving surgery. In the RVOT reconstruction, we routinely performed direct annular measurements with a Hegar dilator, and the decision to preserve the annulus is based on the *Z* score. We determine the cut off value of the pulmonary annular *Z* score as −3.0 and defined annulus preservation as when the remaining annular size was greater than that corresponding to a *Z* score of −3.0.

### Statistical analysis

Data are presented as frequencies, medians with ranges for non-parametric data, or means with SDs for parametric data. Comparison of patient’s characteristics between the groups was performed using Fisher’s exact test or the chi-squared test for categorical variables and the Mann–Whitney *U*-test or Student’s t test for continuous variables. To evaluate and compare the changes in the absolute size and the *Z* score of PVA and PA as time passed, paired T-test and mixed-effect linear regression were used to compare the value for continuous data. The reoperation-free ratio, reintervention-free ratio, or both were analysed with the Kaplan–Meier method, and *P*-value was estimated with a log-rank test. Statistical differences for all tests were defined as significant by a two-sided test with a *P* value less than 0.05.

## RESULTS

### Primary outcomes

Between January 2014 and January 2022, a total of 152 patients diagnosed of TOF-PS underwent staged repair with initial palliation of either ADS or MBTTS, and 110 patients were included in this study. 44(40%) of them had received ADS and MBTTS was performed in another 66(60%) of them. The demographic parameters were comparable between the two groups at the initial palliation. Patients who had ADS as palliation had a significant earlier TOF repair with shorter interstage interval compared to those received MBTTS(*P* < 0.001). The demographic characteristics and surgical profiles are summarized in Table 1.

**Table 1:** Patients’ demographic characteristics and surgical profiles

Variables	MBTTS Group (*n* = 66)	ADS Group (*n* = 44)^a^	*P* value
Sex			
Female	26 (39.4%)	17 (38.6%)	0.936
Male	40 (60.6%)	27 (61.4%)	
Prematurity			
Yes	6 (9.1%)	5 (11.4%)	1.000
No	60 (90.9%)	39 (88.6%)	
Age at initial palliation (months)			
<1	6 (9.1%)	7 (15.9%)	0.292
1–2	10 (15.2%)	9 (20.5%)	
>2	50 (75.7%)	28 (63.6%)	
Weight at initial palliation (kg)	6.6 (4.9,8.7)	5.3 (3.5,7.7)	0.212
Surgical approach			
Median sternotomy	56 (84.8%)	–	–
Thoracotomy	10 (15.2%)	–	–
Distal anastomosis			
Right PA	48 (72.7%)	–	–
Left PA	18 (27.3%)	–	–
Size of shunt, mm			
3	2 (3.0%)	–	–
3.5	11 (16.7%)	–	–
4	46 (69.7%)	–	–
5	7 (10.6%)	–	–
Cardiopulmonary bypass			
Yes	31 (47.0%)	–	–
No	35 (53.0%)	–	–
Interstage period (month)	35.5 (22.4,49.5)	21.4 (11.5,29.9)	<0.001
Age at complete repair (months)			
<24	5 (7.6%)	16 (36.4%)	<0.001
25–36	22 (33.3%)	14 (31.8%)	
37–48	21 (31.8%)	10 (22.7%)	
>48	18 (27.3%)	4 (9.1%)	
Weight at complete repair (kg)	12.5 (10.0,15.0)	9.0 (8.0,10.9)	0.005
Valve-preservation			
Yes	41 (62.1%)	21 (47.7%)	0.149
No	25 (37.9%)	23 (52.3%)	

a Values are reported as numbers (%) and median (min–max) when appropriate.

MBTTS, modified Blalock-Taussig-Thomas shunt; ADS, arterial ductal stent.

Indications for MBTTS were bilateral pulmonary artery hypoplasia in 16(24.3%) patients, severe hypoxemia and marginally small PA necessitating an emergency operation in 45(68.1%) patients, and severe juxtaductal stenosis with unilateral pulmonary artery hypoplasia in 5(7.6%) patients. The type of ductal stents used in this procedure were coronary stent size 3.5–4.5 from Onyx, Avant Garde or Azule. The preferred approach is either axillary artery, femoral artery or femoral vein depending on the anatomy of the ductus arteriosus size 4/5 slender sheath.

**Figure 1:**
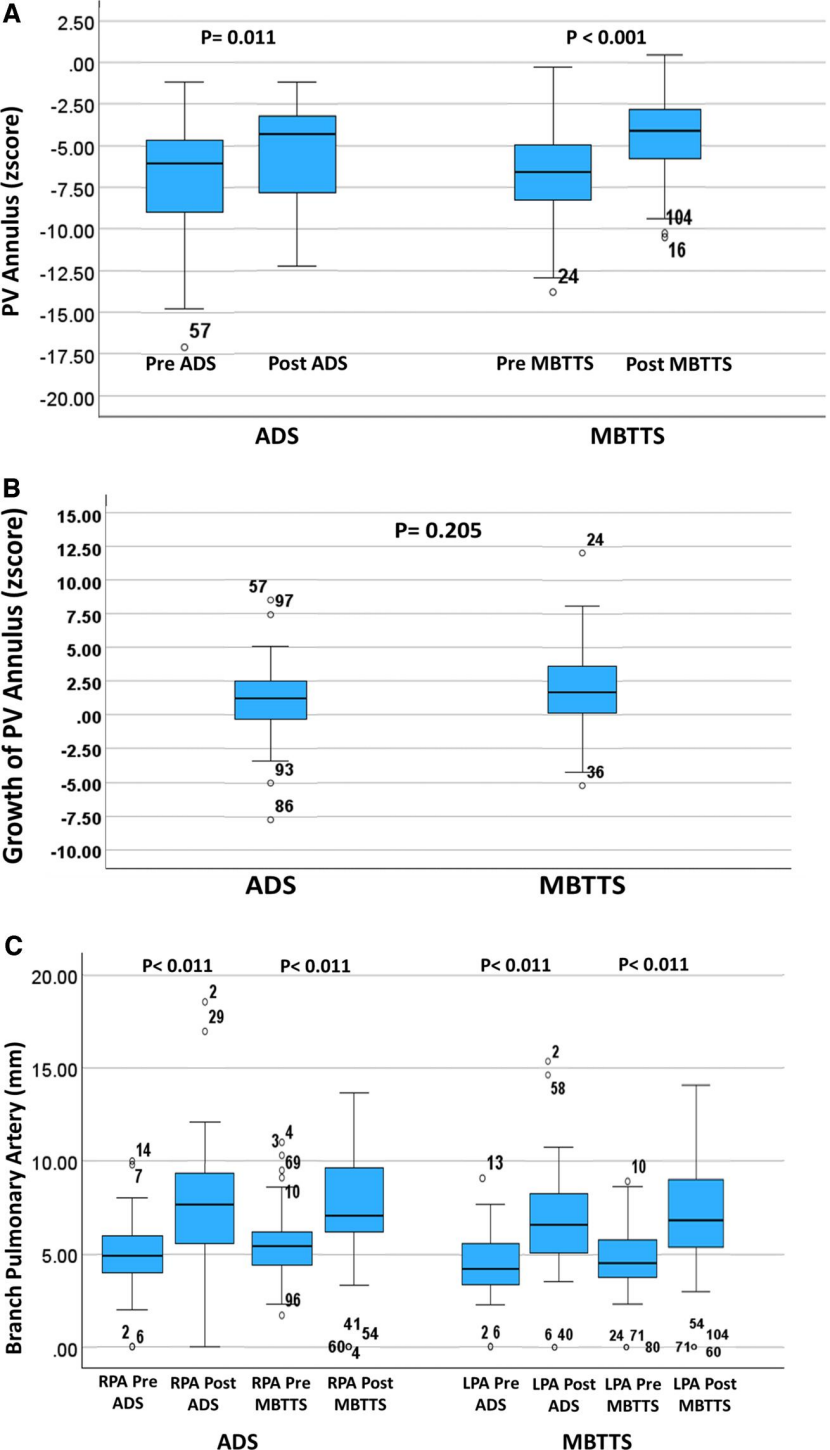
(**A**) PVA growth and (**B**) the increment rate after ADS and MBTTS, (**C**) PA growth (Right and Left PA) after ADS and MBTTS

After the initial palliation, significant growth of PVA was observed in both MBTTS and ADS group as illustrated in Fig. 1A. Although the growth of native PVA was higher following MBTTS than ADS, the difference did not exhibit any statistical significance (*P* = 0.205) as shown in Fig. 1B. Similarly, there was significant increase in branch PA sizes following palliation in both groups(*P* < 0.001), but the inter-group difference was not significant(*P* = 0.230 and *P* = 0.556 for RPA and LPA, respectively) as illustrated in Fig. 1C. Table 2 summarizes the echocardiographic findings before initial palliative procedures and before complete repair of TOF in the 2 groups.

**Figure 2:**
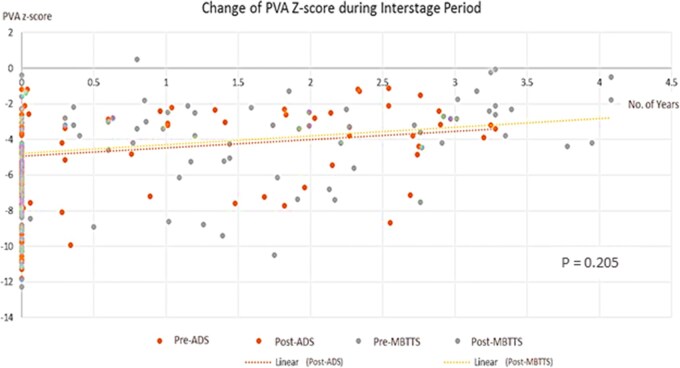
Changes of *Z*-score of PVA according to types of palliation

**Figure 3:**
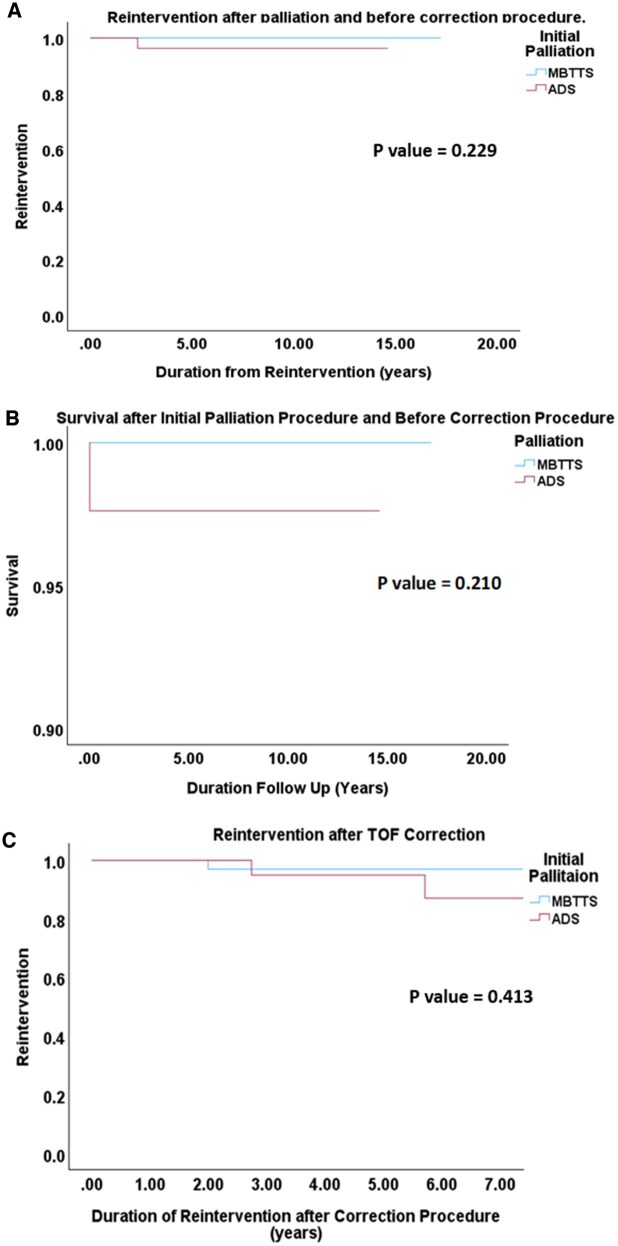
Kaplan–Meier curve for (**A**) freedom from reintervention and (**B**) survival during the interstage period, (**C**) freedom from reintervention after TOF correction

**Table 2:** Patient-related characteristics and echocardiographic findings after initial palliative procedures

Variables	Palliation		Mixed Effect	
	MBTTS group^a^	ADS group^a^	*P* value (between pre and post palliation)	*P* value (between groups)
PVA (Z-score)				
Pre-	−6.6 (−8.3,−4.9)	−6.1 (−9.0,−4.5)	<0.001	0.213
Post-	−4.2 (−5.9,−2.6)	−4.3 (−7.8,−2.4)		0.277
% increase	34.4 (18.6,52.8)	32.9 (16.6,48.5)		0.205
LPA (Z-score)				
Pre-	−0.9 (−3.1,0.5)	−1.5 (−2.8,0.2)	<0.001	0.323
Post-	0.5 (−1.4,1.5)	0.8 (−0.3,1.8)		0.208
% increase	72.3 (44.8,121.5)	93.2 (53.4,147.5)		0.230
LPA (Z-score)				
Pre-	−1.4 (−3.2,−0.2)	−1.7 (−3.5,−0.4)	<0.001	0.697
Post-	0.2 (−0.8,1.5)	−0.2 (−1.6,1.2)		0.382
% increase	83.3 (54.2,132.3)	86.8 (643,147.4)		0.556
Nakata Index				
Pre-	115.4 (68.0,175.4)	100.3 (71.9,158.8)	<0.001	0.744
Post-	232.1 (138.2,426.9)	235.9 (179.8,467.2)		0.593
% increase	121.3 (53.9,177.7))	108.1 (63.9,216.1)		0.780

a Values are reported as median (min–max) when appropriate.

MBTTS, Modified Blalock-Taussig-Thomas shunt; ADS, arterial ductal stent; PVA, pulmonary valve annulus; RPA, right pulmonary artery; LPA, Left Pulmonary Artery.

A total of 62(56.4%) patients were able to undergo pulmonary VSR of TOF successfully, of whom 41(62.1%) patients had MBTTS and 21(47.7%) patients received ADS. Although the native pulmonary annular growth was higher following MBTTS group than ADS group, there was no significant difference in the rate of VSR between the 2 groups(*P* = 0.149). Figure 2 illustrates the time-relevant fashion of PVA growth during the interstage period. We noticed that the patients who successfully underwent VSR had significantly greater growth of PVA and annular *Z*-score after palliation compared to those that did not proceed with VSR regardless of the types of palliative intervention(*P* < 0.001). Furthermore, patients who did not proceed with VSR had smaller initial annular *Z*-score of less than −7 and they failed to exhibit any significant growth of PVA after palliation in both techniques as shown in Table 3. When the patients with severe PS with *Z*-score less than −7 were excluded, the pulmonary annulus could be preserved in 26/37(70.3%) patients in MBTTS, and 18/26(69.2%) patients in ADS.

**Table 3:** The growth of PVA in patients with pulmonary annulus preserved and not preserved during final TOF repair

Variables	Valve preserved N = 62^a^	Valve not preserved N = 48^a^	P value
PVA (Z-score)			
ADS			
Pre-	−5.3 (−6.0,−3.7)	−7.7 (−11.3,−6.1)	0.003
Post-	−3.3 (−4.2,−3.0)	−7.2 (−8.6,−5.8)	<0.001
P-value (within group)	<0.001	0.289	–
MBTTS			
Pre-	−6.2 (−8.0,−4.3)	−7.1 (−8.8,−5.6)	0.096
Post-	−3.2 (−4.1,−2.6)	−6.6 (−8.6,−5.2)	<0.001
P-value (within group)	<0.001	0.480	–

a Values are reported as median (min–max) when appropriate.

MBTTS, modified Blalock-Taussig-Thomas shunt; ADS, arterial ductal stent; PVA, pulmonary valve annulus.

### Secondary outcomes

Table 4 summarizes the immediate postoperative outcomes of patients in both groups. Patients who underwent ADS had significantly shorter hospital stay as compared to MBTTS group (*P* = 0.048). The freedom from reintervention during the interstage period in ADS group and MBTTS group was 97.7% and 100%, respectively, (*P* = 0.229) as illustrated in Fig. 3, with interstage mortality rate of 2.3% and 0%, respectively, (*P* = 0.210). The total follow-up duration was 6.8 ± 3.3 years. Overall, freedom from reintervention after TOF correction at 1, 5 and 7 years was 100%, 95.2% and 90.5% in ADS groups, and 100%, 97.6% and 97.6% in MBTTS group (*P* = 0.413). Among patients who underwent VSR, none of them required reintervention in both the palliation groups. Whereas 3(4.8%) patients (1 from MBTTS group and 2 from ADS group) who had TAP required PR-related reintervention.

**Table 4:** Clinical outcomes after initial palliation procedures

Variables	MBTTS group *N* = 66^a^	ADS group *N* = 44^a^	*P* value
Chest reopen, *n* (%)	1 (1.5%)	1 (2.3%)	1.00
Chylothorax, *n* (%)	5 (7.6%)	–	0.082
Diaphragm paralysis, *n* (%)	1 (1.5%)	–	1.00
ICU stay, days	4.5 (2.0,8.0)	4.0 (2.0,7.0)	0.637
Total hospital stay, days	10.0 (8.0,13.0)	8.0 (5.3,12.0)	0.048
Reintervention before Final TOF correction	0 (0%)	1 (2.3%)	0.229
Interstage mortality	0 (0%)	1 (2.3%)	0.210

a Values are reported as number (%) and median (min–max) when appropriate.

## DISCUSSION

Even though early primary repair is currently the preferred strategy, staged repair with either ADS or MBTTS is still an invaluable palliative option in some high-risk neonates, and not infrequently performed in many institutions [4, 8]. Traditionally, MBTTS has been the preferred choice of palliation for staged repair approach in TOF, but over the last few years, ADS has gained wide acceptance as a reliable alternative to RVOT stent and MBTTS in patients with ductal-dependent pulmonary blood flow [9, 10]. The minimally invasive nature of ADS eliminates the need for thoracotomy or sternotomy and even cardiopulmonary bypass. Besides, this option is currently deemed safer and more effective than palliative surgery in high-risk patients, because it is possible to tailor the shunt magnitude to the patient’s size and pulmonary anatomy [11]. ADS achieves equivalent systemic to pulmonary blood flow to the MBTTS but has advantages in being more distal in the arch and hence theoretically less prone to coronary steal [9, 12]. In our centre, we gradually moved away from RVOT stent in favour of MBTTS and ADS as the presence of stent in RVOT may destroy the pulmonary valve and render subsequent VSR difficult.

Thresholds of PVA size that dictate both short-term and long-term durability are currently not defined [10]. However, we believe that PVA can be preserved in a selective group of patients with favourable pulmonary valve anatomy and acceptable residual PS. Our findings are in support of Blais *et al.* who recommended tolerating moderate residual stenosis in patients with smaller pulmonary annulus undergoing VSR rather than pursuing a TAP procedure, as 30‐year survival and reinterventions were both lower in the VSR group [13]. Preservation of pulmonary valve function without leaving significant residue PS continues to be the biggest challenge in patients undergoing pulmonary VSR. The important question of the best palliative technique to allow for the outgrowth of PVA with the aim of VSR remains. To date, little is known about the impact of ADS on pulmonary annular growth. While other centres have examined the longitudinal growth of the PVA after initial palliation with MBTTS, little contemporary data exists regarding the interstage growth of the PVA and the subsequent rate of VSR among population palliated with ADS. Chong *et al.* has reported the outgrowth of PVA over somatic growth after MBTTS, but preservation of the PVA may not be achieved if the initial PVA is too small [14]. Similarly, Nakashima *et al.* also described the positive impact on the growth of PVA after MBTTS compared to primary repair with an increased tendency of VSR after surgical shunt although the difference was not significant [4]. Kobayashi *et al.* described the outgrowth of PVA after staged repair with MBTTS in symptomatic patients with high number of VSR (46%) [10]. In our study, when looking at the interstage growth of PVA and PA, both the palliative procedures resulted in a significant but similar size increment as well as similar VSR rate in subsequent complete TOF repair. While we believed that a longer interstage duration may result in greater linear growth of PVA in MBTTS, but their differences failed to exhibit any statistical significance, suggesting that the rate of VSR among patients who underwent ADS was comparable to that of MBTTS in staged repair of TOF-PS. However, we noticed that the increment of PVA and its *Z*-score were not significant in infants who had a TAP during TOF repair. Therefore, VSR may still not be achievable if the initial PVA is smaller than *Z*-score of −7 regardless of the types of palliation. In the present study, VSR was successfully performed when the PVA *Z*-score was −3 and greater following the initial palliation.

Freedom from reintervention during the interstage period in both palliation techniques were similar. In our study, only 1 patient required reintervention of the ADS prior to complete TOF correction, with a stent placed at the left PA 4 months after the initial ADS procedure. Furthermore, there was no apparent difference between the two palliative strategies in regard to the clinical outcomes, including the interstage mortality, although patients treated with ADS had a significantly shorter hospital stay. Our findings are similar to Glatz *et al.* who demonstrated comparable rate of death and reintervention between both palliations, with ADS group exhibiting a lower risk of procedural complications, shorter length of ICU stay, and larger, more symmetrical PA before undergoing subsequent surgical repair [9]. During our follow-up, we did not report any patient who required PR-related or RVOT-related reintervention or surgical revision among patients who had received VSR successfully regardless of the types of palliations.

In our centre, we advocate and emphasize on individualized treatment approach rather than upfront primary repair in all TOF-PS patients, which is also advocated by other authors [15, 16]. We have deviated our preference from performance of surgical shunt to ADS in this subset of patients with a marginally small PVA [17]. Based on their clinical conditions, weight at surgery and underlying cardiac anatomy, the decision for the feasibility of surgical shunt or ductal stent is made during multidisciplinary team discussion involving the interventionalist and cardiac surgeons. PDA has been a preferred palliation technique in patients with suitable arterial duct morphology without significant pulmonary artery stenosis. We generally excluded complex PDA anatomy with unusually long ductus with multiple acute bends in different planes (bizarre morphology). The presence of PA stenosis at the site of ductal connection is a class III indication for ductal stenting due to the concern of accelerated stenosis [18]. In our cohort, there was a significant number of patients who received palliative intervention at more than one month of age. The reasons for this delayed intervention are largely multifactorial which include prematurity, low birth weight, late referral and presentation and recurrent infection at referring hospital. Furthermore, most patients presented in the post-natal period with respiratory distress, cyanosis and circulatory collapse, which in a late-presenter, may be due to the presence of a large PDA initially which became constricted with time. All patients were on Prostaglandin E2 infusion prior to intervention to maintain duct patency.

Patients were routinely followed up with clinical assessment, serial echocardiography, cardiac catheterization and CT scan after the initial palliative procedure. We performed final TOF correction in patients who became symptomatic, and the measurements for PVA and branch PA were recorded prior to the TOF correction, which was about 1.5–2 years after the palliation. In our centre, although most of the patients received final corrective surgery within 2 years after the initial palliation but there was a significant number who had late intervention. The delayed intervention was largely due to logistic issues and late referrals. Patients who received ADS became symptomatic earlier compared to those who had MBTTS, and thus final TOF correction was performed on them. However, despite the shorter interstage period for patients who received ADS, the rate of VSR during final TOF correction was still comparable to those of MBTTS.

In our investigation, several key findings emerged in patients received ADS compared to those underwent MBTTS as staged repair of TOF: (i) both palliation strategies successfully increase the PVA and PA significantly and there was no major difference in the rate of increment between the two palliative treatment groups, (ii) ADS demonstrated a shorter total length of hospital stay compared to MBTTS, (iii) the incidence of interstage mortality and rate of reintervention did not differ significantly between the two groups and (iv) incidence of VSR in patients underwent ADS was comparable to patients palliated with MBTTS.

This study is subject to the limitations inherent to a single-institutional, non-randomized retrospective study, and a relatively small cohort size. Although we were able to quantify the outgrowth of the PVA and PA after the palliative procedures, it was difficult to isolate the effect of performance of the palliative procedures from the effects of other factors that may contributed to the growth including the somatic growth of the native PVA and PA. Randomized controlled clinical trials are thus desirable for the accurate assessment of the benefits of a palliative strategy over primary repair in a selected subset of patients. Furthermore, a clear indication based on the pulmonary annulus size (*Z* score) or the determination of the optimal interstage period for sufficient annular growth warrants further study.

## CONCLUSION

ADS is as effective as MBTTS as a palliative procedure in promoting the growth of PVA and branch PA, with shorter hospital stay and low mortality rate. Furthermore, a staged approach to total correction following either of the palliation procedures has been shown to have an increased rate of VSR during corrective surgery in patients with marginally small PVA.

## Data Availability

The data underlying this article are available in the article.

## Notes

Presented at the 38th Annual Meeting of the European Association for Cardio-thoracic Surgery (EACTS), Lisbon, Portugal, October 9–12, 2024.
